# Knowledge, Practice, and Associated Factors of Home-Based Management of Diarrhea among Caregivers of Children Attending Under-Five Clinic in Fagita Lekoma District, Awi Zone, Amhara Regional State, Northwest Ethiopia, 2016 

**DOI:** 10.1155/2017/8084548

**Published:** 2017-08-21

**Authors:** Bogale Kassahun Desta, Nega Tezera Assimamaw, Tesfaye Demeke Ashenafi

**Affiliations:** ^1^Injibara General Hospital, Injibara, Awi Zone, Amhara Regional State, Ethiopia; ^2^Department of Pediatric and Child Health Nursing, School of Nursing, College of Medicine and Health Sciences, University of Gondar, Gondar, Ethiopia

## Abstract

**Introduction:**

In Ethiopia, it is the second cause for clinical presentation among under five-year child population.

**Objective:**

The main aim of this study was to assess knowledge, practice, and associated factors of home-based management of diarrhea among caregivers of children attending the under-five clinic.

**Methods:**

Institution based quantitative cross-sectional study was carried out from March 1, 2016, to April 22, 2016.

**Results:**

Two hundred eight (56.2%) of them had good knowledge and one hundred thirty-nine (37.6%) of them had the good practice of home management of diarrhea, specifically, primary education (AOR: 5.384, 95% CI: 2.008, 14.438), secondary and above education (AOR: 11.769, 95% CI: 3.527, 39.275), daily laborer (AOR: 0.208, 95% CI: 0.054, 0.810), and no information about diarrhea (AOR: 0.139, 95% CI: 0.054, 0.354). Moreover, age range of 25–35 (AOR: 4.091, 95% CI: 1.741, 9.616) and 36–45 (AOR: 3.639, 95% CI: 1.155, 11.460), being single (AOR: 0.111, 95% CI: 0.013, 0.938), being divorced (AOR: 0.120, 95% CI: 0.024, 0.598), illiteracy (AOR: 0.052, 95% CI: 0.017, 0.518), primary education (AOR: 0.143, CI: 0.046, 0.440), and no information about diarrhea (AOR: 0.197, 95% CI: 0.057, 0.685) were significantly associated variables with the outcome variables in multivariate regression.

**Conclusion:**

Caregivers had slightly adequate knowledge but poor practice.

## 1. Background

Diarrhea is defined as the passage of three or more loose or liquid stools per day [1] and is caused by the variety of bacteria, viruses, and parasites [2]. Globally, each year it kills around 7,600,000 children less than five years and 1.7 billion cases are reported every year. These deaths are mainly due to dehydration, which can be prevented by prompt treatment with home-based oral fluids [3]. In Africa, every under-five child experiences five episodes of diarrhea per year, and around 800,000 children die of diarrhea and dehydration each year. Sub-Saharan Africa is the region where high rates of child mortality are reported [4]. In Ethiopia, it is the second cause for clinical presentation among under-five-year child population [5].

Even though there is the significant reduction in morbidity and mortality due to diarrhea, there has to be an improvement in diarrhea case management in homes within the community. However, home-based management of diarrhea among caregivers of under-five children is not adequate especially in developing countries because of the inadequacy of knowledge and practice gaps [6].

As findings from the study conducted in Nepal, Iran, and Nigeria show, the level of knowledge of caregivers on home management diarrhea was inadequate [7–9]. Similarly, the level of practice of caregivers on the home-based management of diarrhea was also poor as determined by studies conducted in Nepal, Iran, Pakistan, and Kenya [8, 10–12]. Few studies in Ethiopia show that the proportion of knowledge and practice of caregivers towards the home management of under-five diarrhea was also found to be inadequate [13].

Beyond the inadequacy of knowledge and practice, there is also some evidence that shows harmful practices like food restriction, breast feeding reduction, and use of traditional and inappropriate medicine which is of unknown effect in managing diarrhea at home performed by caregivers which are also believed to be due to lack of knowledge [14].

As primary caregivers to under-five children in Ethiopia, mothers' knowledge and management skills are important to minimize the effects of morbidity and mortality associated with diarrheal diseases [4]. However, there are no previous studies in the study area that have assessed the level of knowledge and practice of home-based management of diarrhea among caregivers of under-five children. Therefore, this study seeks to determine knowledge, practice, and associated factors of home-based management of diarrhea among caregivers attending under-five clinic Fagita Lekoma district, Northwest Ethiopia, and compare with previous findings in other parts of the country and beyond.

## 2. Materials and Methods

### 2.1. Study Design and Period

An institution based quantitative cross-sectional study was conducted from March 1, 2016, to April 22, 2016.

### 2.2. Study Area

The study area was Fagita Lekoma district, which is found in Awi Zone of Amhara Regional State and is located 472 km far from Addis Ababa, the capital city of Ethiopia, and 100 km far from Bahir Dar, the capital city of Amhara National Regional State. According to the district health office report in 2008 E.C, it has a total population of 156,671, of which the estimated number of under-five children was 21,147. In this district, there are 6 health centers, 3 private clinics, and 27 health posts with 52 health extension workers which give health care services to the population in the district.

### 2.3. Source Population

The source populations for this study were all caregivers attending under-five clinic whose child has diarrhea or episode of it in the past three months prior to the time of study.

### 2.4. Study Population

The study population were all caregivers attending under-five clinic during data collection period and whose child has diarrhea or episode of diarrhea in the past three months prior to the time of study.

### 2.5. Inclusion Criteria

Inclusion criteria were all caregivers whose child has diarrhea or episode of diarrhea in the past three months prior to the time of study.

### 2.6. Exclusion Criteria

exclusion criteria were caregivers who were not volunteers to participate in the study and caregivers whose children are in need of urgent referral.

### 2.7. Sample Size Determination

The sample size for this study was calculated using a single population proportion formula based on the following assumptions:

(1) $$\begin{array}{c} n=\frac{{\left(Z\alpha /2\right)}^{2}\times P\left(1-P\right)}{{\left(d\right)}^{2}}, \end{array}$$

where *n* is minimum sample size required for the study, *d* is margin of error = 0.05, *Zα*/2 is value of standard normal distribution (*Z* = 1.96) with confidence interval of 95%, and *α* is 0.05; *P* = good knowledge = 37.5% [1].

(2) nZα/22×P1−Pd2=1.962×0.3751−0.3750.052=360.

Therefore, by taking the nonresponse rate of 5%, the total sample size required for this study was 360 + 18 = 378.

## 3. Sampling Procedure

A systematic random sampling technique was employed to select the study participants from all health centers of Fagita Lekoma district. Based on the last three-month under-five health care services the average daily flow was calculated. The number of the under-five visits in the second quarter of year of 2008 EC in Addis Kidamie, Finzit, Fagita, Waze, Gezehera, and Chiguali health centers was 1131, 283, 261, 760, 227, and 429, respectively.

**Figure 1:**
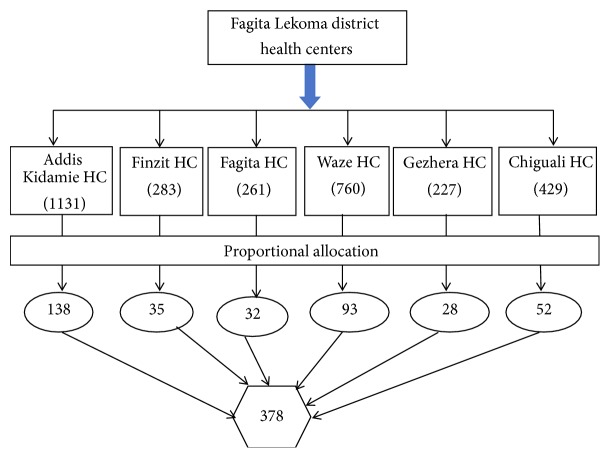
Schematic presentation of the sampling procedure.

Then, the number of actual study participants from each HC was calculated and taken proportionally by using the formula *ni* = (*n*/*N*) × *Ni* (Figure 1).

### 3.1. Variables of the Study

#### 3.1.1. Dependent Variables

  Knowledge of home management of diarrhea
  Practice on home management of diarrhea.

#### 3.1.2. Independent Variables

  Sociodemographic characteristics
  Age of caregiver occupational status
  Sex of caregiver educational status
  Marital status income
  Number of children
  Source of information about diarrhea
  Perceived cause of diarrhea
  Operational definition.

*Adequate Knowledge.* Caregivers who answer above and equal to the mean of knowledge questions are considered as having adequate knowledge.

*Inadequate Knowledge*. Caregivers who answer below to the mean of knowledge questions are considered as having inadequate knowledge.

*Good Practice*. Caregivers who answer above and equal to the mean of practice questions are considered as having the good practice [1].

*Poor Practice*. The study participants who answer below the mean of practice questions are considered as having poor practice.

*Caregivers*. they are mothers or any other caretakers of under-five children.

*Homemade Fluid*. It is recommended homemade fluids easily available and prepared at home like the mixture of salt, sugar, and water, breast milk, Gruels (diluted mixtures of cooked cereals and water), soup, rice water, and so forth.

### 3.2. Data Collection Method and Tool

Structured interview questionnaires were adapted and modified from differently related literature to collect the data on the sociodemographic variables and associated factors [2–8]. It was prepared in English and then translated to Amharic language and then was retranslated into English to check for its consistency. The data were collected through face-to-face interview by trained collectors. Six diploma clinical nurses and one supervisor for data collection activities were recruited.

### 3.3. Data Quality Control

To assure the data quality, the recruited data collectors were trained for half days on the objective, confidentiality of information, the relevance of the study and respondent's right, pretest, informed consent, and techniques of an interview. Before going into data collection, the structured questionnaire was pretested on one private clinic for consistency of understanding the survey tool and modifications were done accordingly. Close supervision was undertaken during data collection.

### 3.4. Data Processing and Analysis

After collection of the data, the responses were coded and entered into Epi-info version 7 software and then exported to SPSS version 20 for analysis. Then, the frequency distribution of dependent and independent variables was worked out. A measure of central tendency was used for various variables of the study and the data were presented with graphics and tables. A crude and adjusted odds ratio from bivariate and multivariate analysis was used to measure the association between dependent and independent variables. Those variables as *p* value <0.2 by the bivariate analysis were taken to binary logistic regression model for further multivariate analysis. Variables at 95% of confidence interval and the *p* value less than 0.05 were considered as statistically significant.

## 4. Results and Discussion

### 4.1. Sociodemographic Characteristics

A total of 378 caregivers were included in this study, of which 370 participants gave a complete response with the response rate of 97.9%. The median age of the participants was 29 with interquartile range of 12. About 96.8% of participants were females while the rest were males. About 99.7% participants were Orthodox and 86.2% of the participants were married. More than half (58.6%) of the participants were illiterates and about 65.4% of mothers were housewives. About 92.4% and 7% of participants were Awi and Amhara, respectively, in their ethnicity. Around 63.8% of mothers have 1-2 children. About 61.6% participants' average monthly income was in a range of 652–1400 ETB. More than one-fourth of participants' children were suffering from diarrhea two weeks before the time of data collection (Table 1).

### 4.2. Knowledge of Caregivers in Home Management of Diarrhea

This study has found that 208 (56.2%) of caregivers had adequate knowledge (95% CI = 51.4, 61.1) while 162 (43.8%) caregivers had inadequate knowledge (95% CI = 38.9, 48.6) regarding home management of diarrhea in under-five children.

Out of all caregivers, two hundred forty-two (65.4%) had correct knowledge of meaning of diarrhea and concerning the signs of diarrhea, 156 (42.2%), 97 (26.2%), and 252 (68.1%) of caregivers said that frequent passage of diarrhea, sunken eyes, and weakness/lethargic are signs of diarrhea in children, respectively. Around 213 (57.6%) of caregivers said that poor hygiene is the cause of diarrhea and about 30.3% caregivers did not know any cause of diarrhea. For about 276 (74.6%) of respondents' source of information regarding diarrhea and its management was health workers, while 12.7% caregivers did not have any information about diarrhea.

Around 91 (24.6%) of respondents did not have any knowledge on the impact of diarrhea disease and about 63.5% of caregivers said that morbidity and mortality are impacts of diarrhea in under-five children.

About 269 (72.7%) of caregivers said that diarrhea is not treatable at home and more than half of the caregivers (51.6%) did not know any type of fluids used for management of diarrhea at home and 28.1% caregivers knew that ORS is one of the fluids used in diarrhea.

Regarding the use of ORS, only 67 (18.1%) caregivers said that it replaces fluid lost during diarrhea but 239 (64.6%) caregivers said it stops diarrhea, while 64 (17.3%) of caregivers did not know the use of ORS. More than two hundred eighty (76.2%) of participants had correct knowledge on preventive ways of diarrhea disease in under-five children, of which 41.6% and 37.3% said that personal hygiene and vaccination are preventive ways of diarrhea.

### 4.3. Practice of Caregivers in Home Management of Diarrhea

This study has revealed that 139 (37.6%) caregivers had the good practice (95% CI = 32.7, 43.2) while 231 (62.4%) caregivers had a poor practice (95% CI = 56.8, 67.3) regarding home management of diarrhea.

Regarding caregivers practice, 20.3% of caregivers did not take any measure, about 71.6% of caregivers took to the health center, and 5.1% of caregivers gave homemade fluids during the episode of diarrhea in their children. On the contrary, about 0.5% and 1.9% of caregivers had given traditional herbs and decreased/stopped feeding pattern during the episode of diarrhea, respectively. Regarding the preparation of homemade fluid, only 35 (9.5%) caregivers used to prepare it, of which only 31 (8.4%) of caregivers had been preparing homemade fluids correctly. More than seventy (73.2%) caregivers had given ORS to their children during the episode of diarrhea of which about 85.4% prepared ORS powder correctly with the recommended amount of water. Regarding methods of ORS giving, about 329 (88.9%) caregivers used cup while 41 (11.1%) of them used the spoon. Concerning frequency, only 60 (16.2%) caregivers administered ORS after each episode of diarrhea while 226 (61.1%) caregivers gave it only whenever the child wants to drink. About 197 (53.2%) caregivers said that they keep the reconstituted ORS for 12 hours while the rest of the mothers said that they keep it for 24-hour duration.

### 4.4. Factors Associated with Knowledge of Caregivers in Home Management of Diarrhea

Age of caregivers, marital status, educational status, occupational status, a source of information, and a number of children were significantly associated with the outcome variable in the bivariate analysis.

In the multivariate analysis, educational status, the occupation of mother, and source of information about diarrhea were significantly associated with maternal/caregiver's knowledge. However, age, marital status, and a number of children were not significantly associated with multivariate analysis.

Specifically, primary education (AOR: 5.384, 95% CI: 2.008, 14.438), secondary and above education (AOR: 11.769, 95% CI: 3.527, 39.275), daily laborer (AOR: 0.208, 95% CI: 0.054, 0.810), and no information about diarrhea (AOR: 0.139, 95% CI: 0.054, 0.354) were significantly associated with the outcome variable knowledge (Table 2).

### 4.5. Factors Associated with Practice of Caregivers in Home Management of Diarrhea

In bivariate analysis, the age of caregivers, marital status, the number of children, the source of information about diarrhea, educational status, and occupational status were significantly associated with the outcome variable practice.

In the multivariate analysis, age, educational status, marital status, and source of information about diarrhea were significantly associated with caregivers practice in the home management of diarrhea. However, a number of children and occupational status were not significantly associated.

Specifically, age range of 25–35 (AOR: 4.091, 95% CI: 1.741, 9.616), age range of 36–45 (AOR: 3.639, 95% CI: 1.155, 11.460), being single (AOR: 0.111, 95% CI: 0.013, 0.938), being divorced (AOR: 0.120, 95% CI: 0.024, 0.598), being illiterate (AOR: 0.052, 95% CI: 0.017, 0.518), primary education (AOR: 0.143, CI: 0.046, 0.440), and no information about diarrhea (AOR: 0.197, 95% CI: 0.057, 0.685) were significantly associated variables with the outcome variable in multivariate regression (Table 3).

### 4.6. Discussion

This study has determined that the good level of knowledge of mothers towards the home management of diarrhea in under-five children was 56.2% with 95% CI (51.4, 61.1). This is relatively higher than the findings from the study conducted in Benishangul Regional State (37.5%) but lower than the findings from the study conducted in Finote Selam town (63.6%) [1, 2]. This difference from Benishangul Region may be due to that caregivers may have been provided more information about diarrhea management in the study area. Additionally, the geographical location may affect access to health care service in Benishangul. In a case of Finote Selam, caregivers have better access for information regarding diarrhea and its home management as the area is urban.

This study showed about 65% of the participants knew the correct definition of diarrhea. This is lower than the findings from the study conducted in India, Pakistan, and Nepal where 90%, 72%, and 67.2% of the participants know the correct definition of diarrhea, respectively [3–5]. This knowledge variation may be due to differences in sociodemographic and access to information about diarrhea. Concerning the cause of diarrhea, the current study has also shown about 30% of the caregivers had not any knowledge and this value is also larger than the study conducted in Pakistan (5%) [3]. Similarly, this discrepancy might be due to a difference in sociodemographic characteristics. This study has demonstrated that the knowledge of mothers on the impacts of diarrhea in children was about 75%. Specifically, about 63% and 20% of participants said that diarrhea causes morbidity/mortality and growth retardation, respectively. But in Nigeria, more than one-third of participants were not knowledgeable concerning the impacts of diarrhea in children [5]. In this study, only 27.3% of caregivers knew that diarrhea can be managed at home. The current study also showed that the awareness of the use of ORS and sugar and salt solution for the home management of diarrhea among participants was 28.1% and 11.4%, respectively. Although 28.1% of caregivers were aware of ORS, only 18.1% of them knew correctly its use in replacing fluid lost during diarrhea. In general, the present study showed about 51.6% of caregivers were aware of all home available fluids used for management of diarrhea. But from the study done in India, ORS was known to almost all respondents.

In addition, the study done in Nigeria shows the awareness of the use of ORS and SSS for the home management of diarrhea was about 90 and 68 percent, respectively.

In another study conducted in India, more than 80% of participants were aware of the use of all home available fluids for the management of diarrhea [4, 6, 7]. The possible reason for this wide discrepancy can be that participants in India and Nigeria may have sufficient information and intensive health education program and there may also be a difference in sociodemographic characteristics including health care service delivery system.

This study identified that caregivers who have primary education and secondary and above education were 5 and 11 times more likely to have good knowledge (OR: 5.384, CI: 2.008, 14.435; OR: 11.769, CI: 3.527, 39.275, resp.) as compared to illiterates. Similarly, educational status of caregivers was significantly associated in studies conducted in India, Iran, and Nigeria [4, 6, 8]. The fact is that as the educational level of caregivers increased, the level of awareness and knowledge increases.

In this study, daily laborers were by 79.2% less likely to have good knowledge (OR: 0.208, CI: 0.054, 0.810) as compared to housewives. This is supported by the study done in Iran [9]. The reason may be that daily laborers could not have the opportunity to get information from different sources like newspaper, television, and radio as the nature of their work makes them busy; but housewives have sufficient time to gain information from different sources.

This study has also shown that caregivers who have no information about diarrhea were by 86.1% less likely to have good knowledge (OR: 0.139, CI: 0.054, 0.354) as compared to their counterparts. This is the general truth that mothers who get information from television, radio newspaper, friends, and so forth about diarrhea know more about home management of diarrhea. Similarly, the source of information was also significantly associated with knowledge in the study conducted in Iran [9].

Regarding practice, this study has shown that the level of practice of caregivers towards the home management of diarrhea was 37.6% with 95% CI (32.7, 43.2). This is in line with the study conducted in Nigeria (33.8%) but lower than the study conducted at West Gojjam, Finote Selam town [2, 6]. The reason is caregivers living in the town have more access and opportunity for information about diarrhea and its management.

This study has revealed that about 20% of caregivers had not taken any measure during diarrhea episodes in their children. This study has also found that about 5% of caregivers gave home fluids and 0.5% of caregivers gave traditional herbs to manage diarrhea. But the findings from the study conducted in Nigeria show that quarters of respondents have used traditional herbs to treat diarrhea for their children [10]. This difference indicates the cultural practice and belief have decreased in our study area as health extension workers may have given health education for the mothers. This study has also shown that about 10% of caregivers used homemade solution, that is, salt-sugar solution (SSS), but only around 8% of them prepared the solution correctly. This is in line with the study conducted in Nigeria [6].

This study has shown that three-fourths of caregivers used ORS while their children started to experience diarrhea, but the study conducted in Nigeria and Vietnam shows more than three-fourths of caregivers had used ORS to treat diarrhea [6, 11]. This variation might be due to sociodemographic differences and other health care service related situations. Concerning feeding practice during diarrhea, the current study showed that more than ninety percent of participants said they continue the usual feeding for the child. But in India and Nigeria, less than half of participants interrupted/decreased/restricted the feeding pattern of their children during diarrhea [12, 13]. This indicates the wrong belief that participants think feeding may exacerbate diarrhea has changed in our study area. The current study showed that about 85% of caregivers used the recommended amount of water for reconstituting ORS powder. The study has also revealed that only 16.2% of mothers practiced the correct frequency of ORS administration, but more than 60% of them administer it to child only whenever the child wants to drink. This is inconsistent with the findings from the study conducted in Pakistan in which about 38% of participants administer ORS to child after the passage of every loose stool [14]. The reason may be due to a difference in sociodemographic characteristics.

Concerning duration, more than half of the caregivers in our study said that they keep the reconstituted ORS for 24-hour duration and discard it after this.

Similarly, the study conducted in South Nigeria shows more than half of participants discard the reconstituted solution of ORS after 24 hours of time [15].

In this study, mothers in an age range of 25–35 and 36–45 years were 4 and 3.6 times more likely to have the good practice (OR: 4.091, CI: 1.741, 9.616 and OR: 3.639, CI: 1.155, 11.460, resp.) as compared to mothers in age range of 15–24 years. This is consistent with the study finding in West Gojjam [2]. However, an age of mothers more than 45 years was not significantly associated with the practice towards the home management of diarrhea. This might be due to that mother who is in age ranges of 25–45 years might have experienced before in managing diarrhea as they may have more children so that the exposure of mothers to diarrhea treatment is high in this age group. But, in the case of elders, they might rely on other cultural and traditional herbs as they are relatively earlier generations in which time access to information and health education was not present.

The present study showed that single and divorced mothers were by 88.9% and 88% less likely to have the good practice (OR: 0.111, CI: 0.013, 0.938 and OR: 0.120, CI: 0.024, 0.598, resp.) as compared to those mothers who were married. The reason may be due to the fact that mothers who were married may have the opportunity to share knowledge and practice from their husband.

This study has indicted mothers who were illiterate and educated till primary school were by 94.8% and 85.7% less likely to practice more (OR: 0.052, CI: 0.017, 0.158 and OR: 0.143, CI: 0.046, 0.440, resp.) as compared to those mothers who had secondary and above educational level. This is supported by the studies conducted at West Gojjam, Iran, India, and Nigeria [2, 6–8]. The justification is that as the educational level of mothers increased, they become more skillful towards diarrhea management.

This study has also identified that mothers who had no information about diarrhea were by 80.3% less likely to have the good practice (OR: 0.197, CI: 0.057, 0.685) compared with their counterparts. This may be due to the fact that mothers who had information about diarrhea have a good opportunity to manage diarrhea at home.

## 5. Limitation of the Study

Since it was a cross-sectional study, it is difficult to know the cause and effect at the same time.

In rare cases, there might be recalled bias among the respondents.

There is shortage of literature for factors associated with knowledge as well as practice.

## 6. Conclusion

Over all, the level of practice was low and there was a slightly higher level of knowledge among caregivers as regards the home management of diarrhea. The level of knowledge was significantly associated with educational status, occupational status, and source of information about diarrhea of mothers. Moreover, the level of practice was significantly associated with the source of information about diarrhea, age, and educational status.

## Figures and Tables

**Table 1:** Sociodemographic characteristics of caregivers attending under-five clinic in the Fagita Lekoma district, Awi Zone, Amhara Regional State, Northwest Ethiopia, 2016 (*n* = 370).

Variables	Frequency (*n* = 370)	Percent
Age		
15–24	93	25.1
25–35	179	48.4
36–45	57	15.4
>45	41	11.1
Sex		
Female	358	96.8
Male	12	3.2
Religion		
Orthodox	369	99.7
Other	1	0.3
Marital status		
Married	319	86.2
Single	19	5.1
Widowed	10	2.7
Divorced	22	5.9
Educational status		
Illiterate	217	58.6
Primary school (1–8)	56	15.1
Secondary school (9–12)	42	11.4
College/university	55	14.9
Occupation		
Housewife	242	65.4
Government employee	62	16.8
Private employee	12	3.2
Daily laborer	35	9.5
Merchant	14	3.8
Farmer	5	1.4
Ethnicity		
Amhara	26	7.1
Awi	342	92.4
Other	2	0.5
Number of children		
1-2	236	63.8
3-4	118	31.9
>4	16	4.3
Diarrhea in the last		
<2 weeks	97	26.2
2 weeks to 1 month	80	21.6
1 month to 2 months	72	19.5
2 months to 3 months	121	32.7

**Table 2:** Factors associated with knowledge in home management of diarrhea among caregivers attending under-five clinic in Fagita Lekoma district, Northwest Ethiopia, 2016.

Variable	Level of knowledge		COR (95% CI)	AOR (95% CI)
	Adequate	Inadequate		
Age				
15–24	53 (57%)	40 (43%)	*4.711 (2.022, 10.976)*	1.253 (0.364, 4.319)
25–35	115 (64.2%)	64 (35.8%)	*6.389 (2.870, 14.221)*	2.740 (1.000, 7.502)
36–45	31 (54.4%)	26 (45.6%)	*4.239 (1.715, 10.476)*	1.950 (0.678, 5.606)
>45	9 (22%)	32 (78%)	1.00	1.00
Marital status				
Married	190 (59.6%)	129 (40.4%)	1.00	1.00
Single	6 (31.6%)	13 (68.4%)	*0.313 (0.116, 0.846)*	0.548 (0.104, 2.893)
Widowed	2 (20%)	8 (80%)	*0.170 (0.035, 0.812)*	0.498 (0.054, 4.580)
Divorced	10 (45.5%)	12 (54.5%)	0.566 (0.237, 1.348)	0.685 (0.169, 2.768)
Educational status				
Illiterate	87 (40.1%)	130 (59.9%)	1.00	1.00
Primary (1–8)	32 (57.1%)	24 (42.9%)	*1.992 (1.099, 3.612)*	*5.384 (2.008, 14.435*)^*∗*^
Secondary and above	89 (91.6%)	8 (8.4%)	*16.624 (7.676, 36)*	*11.769 (3.527, 39.275*)^*∗*^
Occupational status				
Housewife	123 (50.8%)	119 (49.2%)	1.00	1.00
Government emp^*∗*^	61 (98.4%)	1 (1.6%)	*59.016 (8.051, 432.587)*	5.037 (0.516, 49.207)
Private employee	5 (41.7%)	7 (58.3%)	0.691 (0.213, 2.238)	0.245 (0.036, 1.680)
Daily laborer	10 (28.6%)	25 (71.4%)	*0.387 (0.178, 0.840)*	*0.208 (0.054, 0.810*)^*∗*^
Merchant	9 (64.3%)	5 (35.7%)	1.741 (0.567, 5.347)	0.441 (0.113, 1.728)
Farmer	2 (40%)	3 (60%)	0.644 (0.243, 3.952)	0.379 (0.186, 1.962)
Number of children				
1-2	149 (63.1%)	87 (36.9%)	1.00	1.00
3-4	55 (46.6%)	63 (53.4%)	*0.510 (0.326, 0.798)*	1.141 (0.560, 2.322)
>4	4 (25%)	12 (75%)	*0.195 (0.061, 0.622)*	0.609 (0.138, 2.688)
Information about diarrhea				
No information	6 (12.8%)	41 (87.2%)	*0.088 (0.036, 0.213)*	*0.139 (0.054, 0.354*)^*∗*^
Have information	202 (62.5%)	121 (37.5%)	1.00	1.00

*NB*. emp^*∗*^ (employee), enter method in logistic regression model was used to select the variable; the model was adequate with *p* value 0.830 (Hosmer and Lemeshow test for goodness of fit); *∗* indicates significantly associated variables (*p* value <0.05).

**Table 3:** Factors associated with practice of home management of diarrhea among caregivers attending under-five clinic in Fagita Lekoma district, Northwest Ethiopia, 2016.

Variable	Practice		COR (95% CI)	AOR (95% CI)
	Good	Poor		
Age				
15–24	32 (34.4%)	61 (65.6%)	1.00	1.00
25–35	81 (45.3%)	98 (54.7%)	1.576 (0.937, 2.648)	*4.091 (1.741, 9.616*)^*∗*^
36–45	22 (38.6%)	35 (61.4%)	1.198 (0.605, 2.374)	*3.639 (1.155, 11.460*)^*∗*^
>45	4 (9.8%)	37 (90.2%)	*0.206 (0.067, 0.630)*	1.568 (0.343, 7.174)
Marital status				
Married	131 (41.1%)	188 (58.9%)	1.00	1.00
Single	2 (10.5%)	17 (89.5%)	*0.169 (0.038, 0.743)*	*0.111 (0.013, 0.938*)^*∗*^
Widowed	1 (10%)	9 (90%)	0.159 (0.020, 1.274)	0.157 (0.012, 2.036)
Divorced	5 (22.7%)	17 (77.3%)	0.422 (0.152, 1.173)	*0.120 (0.024, 0.598*)^*∗*^
Educational status				
Illiterate	43 (19.8%)	174 (80.2%)	*0.053 (0.028, 0.098)*	*0.052 (0.017, 0.158*)^*∗*^
Primary (1–8)	16 (28.6%)	40 (71.4%)	*0.085 (0.039, 0.186)*	*0.143 (0.046, 0.440*)^*∗*^
Secondary and above	80 (82.3%)	17 (17.7%)	1.00	1.00
Occupational status				
Housewife	67 (27.7%)	175 (72.3%)	1.00	1.00
Government employee	56 (90.3%)	6 (9.7%)	*24.378 (10.033, 59.231)*	2.392 (0.661, 8.652)
Private employee	3 (25%)	9 (75%)	0.871 (0.229, 3.314)	2.849 (0.352, 23.084)
Daily laborer	5 (14.3%)	30 (85.7%)	0.435 (0.162, 1.169)	0.973 (0.229, 4.128)
Merchant	8 (57.1%)	6 (42.9%)	*3.483 (1.165, 10.413)*	2.017 (0.512, 7.947)
Farmer	3 (60%)	2 (40%)	3.917 (0.978, 11.831)	1.108 (0.459, 4.691)
Number of children				
1-2	104 (44.1%)	132 (55.9%)	1.00	1.00
3-4	33 (28%)	85 (72%)	*0.493 (0.306, 0.794)*	1.235 (0.575, 2.653)
>4	2 (12.5%)	14 (87.5%)	*0.181 (0.040, 0.816)*	0.764 (0.127, 4.585)
Information about diarrhea				
No information	3 (6.4%)	44 (93.6%)	*0.094 (0.029, 0.308)*	*0.197 (0.057, 0.685*)^*∗*^
Have information	136 (42.1%)	187 (57.9%)	1.00	1.00

*NB*. Enter method in logistic regression model was used to select the variable; the model was adequate with *p* value 0.984 (Hosmer and Lemeshow test for goodness of fit); *∗* indicates significantly associated variables (*p* value <0.05).
